# Ciprofibrate increases cholesteryl ester transfer protein gene expression and the indirect reverse cholesterol transport to the liver

**DOI:** 10.1186/1476-511X-8-50

**Published:** 2009-11-23

**Authors:** Eliete JB Bighetti, Patrícia R Patrício, Andrea C Casquero, Jairo A Berti, Helena CF Oliveira

**Affiliations:** 1 Physiology and Biophysics Division, Biology Institute, State University of Campinas, Campinas, SP, Brazil

## Abstract

**Background:**

CETP is a plasma protein that modulates atherosclerosis risk through its HDL-cholesterol reducing action. The aim of this work was to examine the effect of the PPARα agonist, ciprofibrate, on the CETP gene expression, in the presence and absence of apolipoprotein (apo) CIII induced hypertriglyceridemia, and its impact on the HDL metabolism.

**Results:**

Mice expressing apo CIII and/or CETP and non-transgenic littermates (CIII, CIII/CETP, CETP, non-Tg) were treated with ciprofibrate during 3 weeks. Drug treatment reduced plasma triglycerides (30-43%) and non-esterified fatty acids (19-47%) levels. Cholesterol (chol) distribution in plasma lipoprotein responses to ciprofibrate treatment was dependent on the genotypes. Treated CIII expressing mice presented elevation in VLDL-chol and reduction in HDL-chol. Treated CETP expressing mice responded with reduction in LDL-chol whereas in non-Tg mice the LDL-chol increased. In addition, ciprofibrate increased plasma post heparin lipoprotein lipase activity (1.3-2.1 fold) in all groups but hepatic lipase activity decreased in treated CETP and non-Tg mice. Plasma CETP activity and liver CETP mRNA levels were significantly increased in treated CIII/CETP and CETP mice (30-100%). Kinetic studies with ^3^H-cholesteryl ether (CEt) labelled HDL showed a 50% reduction in the ^3^H-CEt found in the LDL fraction in ciprofibrate treated compared to non-treated CETP mice. This means that ^3^H-CEt transferred from HDL to LDL was more efficiently removed from the plasma in the fibrate treated mice. Accordingly, the amount of ^3^H-CEt recovered in the liver 6 hours after HDL injection was increased by 35%.

**Conclusion:**

Together these data showed that the PPARα agonist ciprofibrate stimulates CETP gene expression and changes the cholesterol flow through the reverse cholesterol transport, increasing plasma cholesterol removal through LDL.

## Background

Apolipoproteins (apo) CIII are small proteins mainly found on the surface of apoB containing lipoproteins (including VLDL, IDL and LDL), which strongly affect their metabolism. They inhibit catabolism of these lipoproteins by lipoprotein-lipase (LPL) and retard their clearance from plasma by decreasing their affinity for hepatic lipoprotein receptors [1-3]. The ability of apo CIII in inducing hypertriglyceridemia was directly demonstrated in transgenic mice over-expressing this protein [4]. Growing evidences have linked apo CIII concentration in VLDL and LDL to coronary heart disease (CHD) [5,6]. Furthermore, Lee et al. [7] have reported that LDL containing apo CIII strongly predicts coronary events in diabetic patients independently of other lipid concentrations.

Cholesteryl ester transfer protein (CETP) promotes the transfer of cholesteryl esters (CE) from high density lipoprotein (HDL) to the apoB-containing lipoprotein particles, which are subsequently cleared from the circulation by the liver. Thus, CETP plays a critical role in the intravascular remodeling and recycling of HDL particles [8]. Inhibitors of CETP have been tested in human subjects and shown to markedly increase the concentration of HDL cholesterol while decreasing that of LDL cholesterol and apo B [9,10], but the impact of CETP mediated lipid transfer reactions on atherogenesis remains controversial [11]. A large clinical trial with the CETP inhibitor torcetrapib has been prematurely interrupted due to elevation in mortality in the torcetrapib treated group [10]. Human subjects with genetic CETP deficiency and high levels of HDL-cholesterol (>60 mg/dL) showed reduced risk of CHD, whereas CETP-deficient subjects whose HDL levels were moderately increased (40-60 mg/dL) presented higher risk of CHD [12]. Studies in CETP transgenic mice have provided mixed results. The effects of CETP expression in this species can be neutral, pro- or antiatherogenic depending upon the metabolic context [13-17].

Fibrates are drugs widely used to correct hypertriglyceridemia. They exert their effect by activating specific transcription factors called peroxisome proliferator-activated receptors (PPARs), which in turn alter the transcription of several genes encoding for proteins that control lipoprotein metabolism. Thus, fibrates decrease plasma triglyceride (TG) concentrations by activating enzymes responsible for β-oxidation of long chain fatty acids, reducting TG synthesis, activating lipoprotein lipase and repressing the apo CIII expression [18,19]. Fibrates can also alter the metabolism of HDL associated proteins. In humans, treatment with fibrates is associated with an increase in plasma HDL-cholesterol and apo AI concentrations [20]. However, in rodents, fibrates decrease apo AI mRNA and plasma concentrations [21] due to a polymorphism in the PPAR responsive elements in the apo AI gene [22]. Bouly et al. [23] showed that fenofibrate treatment decreased the activity of lecithin cholesterol acyl transferase (LCAT) and increased phospholipid transfer protein (PLTP), while no changes were observed hepatic lipase (HL) activity in mice. Regarding the plasma levels of CETP, varying results were obtained in fibrate treated hyperlipidemic subjects, as follow: CETP decreased with bezafibrate and fenofibrate [24-27], or did not change with gemfibrozil, fenofibrate and bezafibrate [25,28-31], or increased with gemfibrozil [32]. In CETP transgenic mice, two studies reported conflicting results, estimulation [33] and repression [34] of CETP expression by fenofibrate. One type of fibrate largely employed in clinical interventions, ciprofibrate, has not been evaluated regarding its effect on CETP levels. Since CETP is a potent modulator of HDL levels, it is relevant to clarify whether fibrates affect its gene expression and impact HDL metabolism.

Mouse models genetically modified to express specific human genes provide a good opportunity to re-examine the effect of established drugs and to characterize new sites and mode of action. Thus, the aims of the present work were to examine the effect of ciprofibrate treatment on the CETP gene expression, in the presence and absence of apo CIII induced hypertriglyceridemia, and its impact on the HDL metabolism. For this purpose we used transgenic mice over expressing human apo CIII and CETP genes driven by their natural promoters.

## Materials and methods

### Animal procedure

All animal protocols were approved by the university's Committee for Ethics in Animal Experimentation (CEEA/UNICAMP). The mice were housed in a temperature-controlled room on a 12 h light-dark cycle and had free access to food (standard rodent chow; Nuvital, Colombo, Brazil) and water. Hemizygous human CETP transgenic mice, line 5203, C57BL6/J background, expressing a human CETP minigene under the control of its natural flanking sequences [35] and mice overexpressing the human apolipoprotein CIII, line 3707 [36] were derived from Dr. Alan R. Tall's colony (Columbia University, New York, NY). The pups' tail tips were utilized for screening for the presence of the CETP gene promoter by polymerase chain reaction (PCR) DNA amplification of the -538 to -222 CETP promoter region (GeneBank U71187). Tail blood was also drawn for determining plasma CETP activity [37]. Apo CIII transgenic mice were screened by the level of plasma triglycerides: transgenic > 300 mg/dL and non-transgenic < 100 mg/dL. CETP and apoCIII transgenic mice were crossbred to generate 4 genotypic groups: CETP, CIII, CETP/CIII and non-transgenic. Male and female littermates, 10-14 weeks of age, were studied after 3 weeks of treatment (v.o.) with vehicle (2% arabic gum) or ciprofibrate (10 mg/Kg). Mice fasted overnight were anesthetized using ketamine (50 mg/kg, i.p., Parke-Davis, São Paulo, Brazil) and xylazine (16 mg/kg, i.p., Bayer S.A., São Paulo, Brazil) and killed by exsanguination through the retroorbital plexus. Blood samples were centrifuged at 2,500 × *g *at 4°C for 10 min. Aliquots of plasma were stored at -80°C until analyses.

### Biochemical analyses in plasma

Lipoproteins from pooled or individual plasmas of mice were separated by fast protein liquid chromatography (FPLC) using a HR10/30 Superose 6 column (Amersham-Pharmacia Biotech., Uppsala, Sweden) as described previously [38]. Total cholesterol and triacylglycerols (Chod-Pap, Roche Diagnostic GmbH., Mannheim, Germany) and plasma free fatty acids (Wako Chemical, Neuss, Germany) were determined by enzymatic-colorimetric methods according to the manufacturer's instructions. Plasma glucose concentrations were determined by the glucose oxidase method using the Merck Diagnostic-Biotrol^® ^Kit (Chennevières-les-Louvres, France) according to the instructions. Insulin concentrations were determined by radioimmunoassay as described previously [39] using rat insulin as standard.

### Intravascular lipases activities

Total lipase activity was determined according to Ehnholm & Kuusi [40]. Briefly, overnight fasted mice plasmas, obtained before (basal) and 10 minutes after heparin I.V. injection (100 U/Kg body weight), were incubated with a ^3^H-triolein/arabic gum substrate (9,10 ^3^H (N)-triolein, New England Nuclear, Boston, MA) in 0.2 M Tris-HCl buffer, pH 8.5, 37°C, during 1 hour. Hepatic lipase (HL) activity was determined in paralell tubes where the lipoprotein lipase (LPL) was inhibited by 2 M NaCl. The hydrolyzed labeled free fatty acids were extracted with methanol/chloroform/heptane (1.4 : 1.25 : 1), 0.14 M K_2_CO_3_/H_3_BO_3_, pH 10.5, dried under N_2_, and their radioactivity was determined in a liquid scintillation solution in a LS6000 Beckman Beta Counter. The LPL activity was calculated as the difference between the total and the hepatic lipase activities.

### Cholesteryl ester transfer protein activity (exogenous assay)

CETP activity which reflects the plasma CETP concentration [41] was measured using an exogenous substrates assay as previously described [37]. Briefly, a mixture of human VLDL and LDL protein (100 μg) were incubated with 10,000 dpm of human HDL_3 _labeled with [^14^C]-cholesteryl ester (CE) [42] and 5 μl of mice plasma as the source of CETP in a final volume of 100 μl. Blanks were prepared with tris/saline/EDTA buffer (10 mM/140 mM/1 mM), pH 7.4, and negative controls with non-transgenic mice plasma. The incubations were carried out at 40°C for 2 hours. The apo B containing lipoproteins were precipitated using a mixture of 1.6% dextran sulfate/1 M MgCl_2 _solution (1:1) and the radioactivity was measured in the remaining supernatant in a scintillation solution Ultima Gold (Eastman Kodak Co., NY) in a LS6000 Beckman Beta Counter. The % CE transferred from [^14^C]-CE-HDL to VLDL+LDL was calculated as: (dpm in the blank tube - dpm in the plasma sample/dpm in the blank tube) × 100.

### Reverse transcriptase-polymerase chain reaction (RT-PCR) for liver CETP mRNA

Total liver RNA was extracted from ~200 mg of tissue using Trizol reagent (Invitrogen, Grand Island, NY). The integrity of the RNA was checked in tris-borate 1.2% agarose gels stained with ethidium bromide. The amount and purity of the RNA were determined by OD readings at 260 and 280 nm (Gene Quant, Amersham-Pharmacia Biotech, Uppsala, Sweden). Genomic DNA contamination was excluded by running a PCR on the RNA samples. cDNA was obtained from 1 μg of total RNA by reverse transcription using 150 ng of random primers, 10 mM of dNTPs and 200 U of Moloney murine leukemia virus reverse transcriptase (Superscript II; Invitrogen, Grand Island, NY) in a final volume of 20 μl. The tubes were incubated for 60 min at 42°C followed by 15 min at 70°C to inactivate the enzyme. For PCR, a pair of primers was designed to amplify the region spanning part of exon 7 and exons 8 to 14 of the human CETP cDNA, which generated a 668 bp fragment. PCR mixtures consisted of 1 μl of cDNA, 15 pmol of primers, 2.5 U Taq polymerase and 200 μM of dNTPs in a final volume of 25 μl. A initial denaturation at 94°C for 4 min was followed by 28 cycles of 1 min at 94°C, 1.5 min at 58°C and 2 min at 72°C in a Gene Amp PCR System 9700 (Perkin-Elmer, Norwalk, CT). In parallel tubes, the same cDNA was amplified with primers (10 pmol) for rat β-actin cDNA, which generated a 533 bp fragment as an internal standard for the samples. The PCR conditions for β-actin were 2 min at 94°C followed by 23 cycles of 30 s at 94°C, 30 s at 57°C and 45 s at 72°C. The PCR products were separated by electrophoresis on 1.8% agarose gels and the DNA was visualized by ethidium bromide staining. The band intensities were determined by digital scanning and quantitation using Scion Image analysis software (Scion Corp., Frederick, USA).

### Kinetic studies with labeled HDL

HDL was labeled with ^3^H-cholesteryl oleoyl ether (Amersham Biosciences, Buckinghamshire, England) as described by Oliveira and Quintão [42]. More than 95% of ^3^H-HDL was recovered in the cholesteryl ester band after extraction and thin layer chromatography. Labeling efficiency was about 30%. Labeled HDL was filtered in 0.22 μm Millipore membrane and used freshly. Awaken mice received an intraperitoneal (*ip*) injection of ^3^H-HDL (1 × 10^6 ^dpm). Blood samples (50 μl) were obtained by the tail tip at 0.5, 1, 2, 3, 4 and 6 hours after *ip *injection. Cholesterol radioactivity was determined in the supernatant of apo B lipoprotein precipitated plasma samples in a liquid scintillation solution in a beta counter (Beckman LS 6000 TA). Area under the radioactivity versus time curve was determined as previously reported [43]. Final plasma samples obtained by retro orbital bleeding of anesthetized mice were submitted to FPLC, as described above, to determine radioactivity distribution in the lipoprotein fractions.

## Results

Mice expressing apo CIII and/or CETP and non-transgenic littermates (CIII, CIII/CETP, CETP, non-Tg) were treated with ciprofibrate during 3 weeks. After this period, fibrate treatment did not change body weight but relative liver weight increased significantly (48-69%, p < 0.05) in all four genotypic groups of mice, an expected effect for PPARα agonists [18]. Liver and muscle fat and glycogen content and gonadal adipose tissue weight were not affected by ciprofibrate treatment (data not shown).

Plasma lipid, glucose and insulin concentrations before and after ciprofibrate treatment are shown in Table 1. Cipro treatment reduced TG (30-43%) and FFA (19-47%) levels, did not change significantly the total cholesterol and insulin levels, and increased (11-43%) glycemia. The 10-40% elevation in glucose concentrations was confirmed in a shorter 2-week period of treatment (data not shown).

**Table 1:** Effect of ciprofibrate on the fasting plasma levels of lipids and glucose.

Mice groups		Triglycerides (mg/dL)	Cholesterol (mg/dL)	Free Fatty Acids (mmol/L)	Glucose (mg/dL)	Insulin (ng/ml)
CIII	control	429 ± 134 (12)	124 ± 32 (12)	2.9 ± 0.5 (7)	70 ± 15 (6)	0.7 ± 0.2 (6)
	treated	288 ± 140 (8)^a^	102 ± 39 (10)	2.2 ± 0.8 (10)	103 ± 24 (6)^a^	0.6 ± 0.2 (6)
						
CIII/CETP	control	320 ± 141 (10)	100 ± 23 (10)	3.5 ± 0.8 (10)	82 ± 23 (10)	0.7 ± 0.2 (9)
	treated	226 ± 142 (9)	92 ± 20 (8)	2.4 ± 0.8 (8)	91 ± 20 (8)^a^	0.6 ± 0.3 (6)
						
CETP	control	86 ± 37 (10)	79 ± 27 (11)	1.7 ± 0.5 (12)	81 ± 27 (11)	0.6 ± 0.2 (8)
	treated	49 ± 7 (9)^b^	78 ± 23 (11)	0.9 ± 0.3 (10)^c^	91 ± 23 (11)	0.5 ± 0.2 (12)
						
non-Tg	control	93 ± 38 (10)	74 ± 32 (10)	1.6 ± 0.6 (13)	69 ± 32 (10)	0.4 ± 0.2 (6)
	treated	56 ± 12 (9)^b^	93 ± 25 (10)	1.3 ± 0.4 (8)	99 ± 27 (10)^a^	0.6 ± 0.2 (6)

Normolipidemic (non-transgenic and CETP transgenic) and hypertriglyceridemic (CIII and CIII/CETP transgenic) mice were treated with ciprofibrate or vehicle (control) during 3 weeks. Mean ± SD (n). ^a ^*p *< 0.05; ^b ^*p *< 0.01; ^c ^*p *< 0.001 *vs*. control.

Cholesterol (chol) distribution in plasma lipoproteins is shown in Table 2. The response to ciprofibrate treatment was markedly dependent on the genotypes. Treated CIII mice presented elevation in VLDL-chol (88%) and reduction in HDL-chol (45%), resulting in a 3-fold increase in the (V+LDL)/HDL ratio. CIII/CETP mice responded to ciprofibrate with increases in VLDL (178%) and reductions in LDL (61%) and HDL (39%). This also led to a 2-fold increase in the (V+LDL)/HDL ratio. In ciprofibrate treated CETP mice, there was a reduction in LDL-chol fraction (29%) while in non-transgenic mice the LDL-chol increased (122%). In both, CETP and non-Tg, there were no changes in the (V+LDL)/HDL ratio.

**Table 2:** Variation of cholesterol distribution in plasma lipoproteins after ciprofibrate treatment.

mice groups		VLDL mg/dL (%)	LDL mg/dL (%)	HDL mg/dL (%)	VLDL+LDL HDL
CIII	control	30.6 ± 6.0 (24.6)	16.8 ± 1.0 (13.6)	76.6 ± 6.0 (61.8)	0.6
	treated	47.1 ± 2.4 (46.2)^a^	20.4 ± 6.0 (20.0)	34.4 ± 9.5 (33.7)^a^	2.0
					
CIII/CETP	control	20.8 ± 5.0 (20,8)	26.2 ± 8.0 (26.2)	52.9 ± 10.1 (52.9)	0.9
	treated	53.3 ± 8.4 (57.9)^a^	9.3 ± 2.0 (10.1)^b^	29.3 ± 8.0 (32.0)^a^	2.1
					
CETP	control	1.7 ± 0.2 (2.2)	12.8 ± 2.1 (16.2)	64.5 ± 4.3 (81.6)	0.2
	treated	1,8 ± 0,1(2.3)	8.9 ± 0.4 (11.5)^a^	67.3 ± 4.8 (86.2)	0.2
					
non-Tg	control	3.6 ± 0.8 (4.9)	6.5 ± 2.8 (8.8)	63.8 ± 6.2 (86.3)	0.2
	treated	3.3 ± 0.4 (3.6)	16.7 ± 2.2 (18.0)^a^	72.9 ± 5.0 (78.4)^a^	0.3

Normolipidemic (non-transgenic and CETP transgenic) and hypertriglyceridemic (CIII and CIII/CETP transgenic) mice were treated with ciprofibrate or vehicle (control) during 3 weeks. Mean ± SD, n = 6. Data were calculated as the area under VLDL, LDL and HDL peaks of the FPLC profile. Relative cholesterol distribution (%) is given in parenthesis. Mann Whitney test for treated vs control: ^a ^p < 0.05, ^b ^p < 0.005.

As expected, ciprofibrate treatment increased plasma post heparin LPL activity (1.3-2.1 fold) in all groups except in CIII/CETP group (Table 3). Hepatic lipase was not altered in apo CIII expressing mice (CIII and CIII/CETP) but decreased significantly in normotriglyceridemic mice (CETP and non-Tg) (Table 3).

**Table 3:** Effect of ciprofibrate treatment on the lipoprotein lipase (LPL) and hepatic lipase (LH) plasma activities.

Mice groups		LPL (nmol FFA/mL/h)	LH (nmol FFA/mL/h)
CIII	control	6231 ± 279 (5)	2732 ± 2981 (5)
	treated	11990 ± 610 (5)^c^	3829 ± 1595 (5)
			
CIII/CETP	control	7188 ± 1244 (5)	3018 ± 550 (5)
	treated	7620 ± 1993 (6)	3835 ± 611 (5)
			
CETP	control	3906 ± 2099 (7)	2863 ± 951 (5)
	treated	8378 ± 1146 (6)^b^	2079 ± 328 (6)^a^
			
non-Tg	control	6724 ± 1126 (5)	3249 ± 806 (7)
	treated	8957 ± 2984 (5)^a^	2706 ± 994 (5)^a^

Normolipidemic (non-transgenic and CETP transgenic) and hypertriglyceridemic (CIII and CIII/CETP transgenic) mice were treated with ciprofibrate or vehicle (control) during 3 weeks. Mean ± SD (n). ^a ^*p *< 0.05; ^b ^*p *< 0.01; ^c ^*p *< 0.001 *vs*. control.

**Figure 1:**
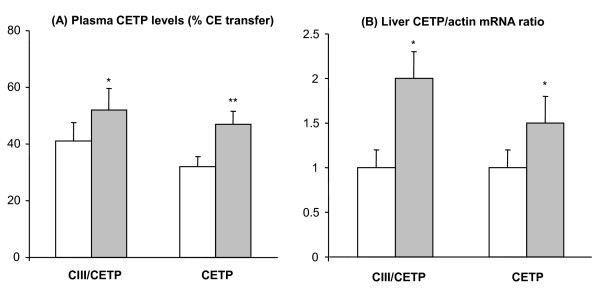
**Ciprofibrate increases CETP plasma levels and liver mRNA**. Plasma CETP levels was measured as cholesteryl ester (CE) transfer rates from exogenous labeled HDL to V+LDL acceptors (A). Panel B shows the relative hepatic CETP mRNA levels. Dark and open bars correspond to ciprofibrate treated and control CETP expressing mice, respectively. Mean ± SE, n = 7-10. Student t test: *P < 0.05; **p < 0.005.

The effects of ciprofibrate on the CETP plasma levels and liver mRNA abundance are shown in figure 1. CIII/CETP and CETP mice treated with ciprofibrate showed 30 and 50% increase in plasma CETP activity, respectively, and 100 and 50% increase in relative liver mRNA levels, respectively.

**Figure 2:**
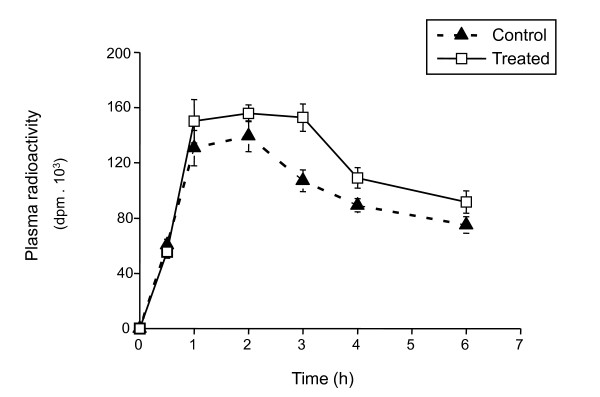
**Ciprofibrate treatment delays plasma removal of ^3^H-cholesteryl ether (^3^HCet) derived from HDL**. Plasma kinetic of ^3^HCet labeled HDL injected intraperitoneally into ciprofibrate treated and control CETP transgenic mice was followed through 6 hours. Each point represents the average of radioactivity in plasma from 6 mice per group. The area under the radioactivity versus time curve was 23% larger for the treated than for control CETP mice (704 ± 39 vs. 572 ± 55 × 10^3 ^dpm.h, respectively, p < 0.05).

We hypothesized that higher plasma CETP and lower HL activities in fibrate treated CETP mice could change the cholesterol flow through the reverse cholesterol transport, increasing indirect removal through LDL and decreasing direct removal through HDL liver uptake. Thus, ^3^H-cholesteryl ether (CEt) labelled HDL was injected into ciprofibrate treated and control CETP mice and followed plasma radioactivity up to 6 hours. Results are shown in figure 2. The area under the radioactivity versus time curve was 23% larger for the treated than for control CETP mice (704 ± 39 vs. 572 ± 55 × 10^3 ^dpm.h, respectively, p < 0.05), indicating that greater amount of HDL remained in the plasma of treated mice during this 6 h-period. Radioactivity distribution in plasma lipoprotein fractions at 6 h after the ^3^H-HDL injection was determined by FPLC (Table 4). Ciprofibrate treated CETP mice presented significantly higher levels of labeled HDL (16%) and a marked reduction (56%) of ^3^H-CEt remaining in LDL fraction (p < 0.005) when compared to control CETP mice. This means that ^3^H-CEt transferred from HDL to LDL was more efficiently removed from the plasma of fibrate treated than from control CETP mice and explains the LDL-cholesterol mass reduction observed in treated CETP mice (Table 2). To confirm this interpretation, we measured liver uptake of 3H-CEt 6 hours after labeled HDL injection. Indeed, whole liver ^3^H-CEt content was significantly higher in ciprofibrate treated than in control CETP mice (36614 ± 4925 vs. 23946 ± 5745 dpm/g, p < 0.05).

**Table 4:** Ciprofibrate treatment increases LDL- and decreases HDL- cholesterol plasma removal. Relative ^3^H-cholesteryl ether distribution (%) in plasma lipoproteins six hours after the injection of ^3^H-HDL into ciprofibrate treated or control CETP transgenic mice.

Groups	VLDL	LDL	HDL
control (n = 5)	6.4 ± 1.8	18.3 ± 3.9	75.2 ± 4.6
			
treated (n = 5)	5.2 ± 1.7	7.9 ± 1.8^b^	86.9 ± 6.4^a^

Mean ± SD. Plasma samples were fractionated by FPLC. Treated vs. control ^a ^p < 0.05; ^b ^p < 0.005.

## Discussion

Fibrates have been extensively and successfully used as hypotriglyceridemic drugs. However, differential efficiency and lipid responses have been found, which are probably related to the type of fibrates and to genetic variation in treated patients. A paradoxal increase in total or LDL-cholesterol in fibrate treated subjects is not very uncommon [44-46].

In this work we showed that some important changes in lipoprotein profile responses to ciprofibrate treatment are largely dependent on the baseline genotype-phenotype, specifically on the high levels of apoCIII and CETP. Fibrate treatment reduced TG levels in both hypertriglyceridemic (CIII and CIII/CETP) and normotriglyceridemic (CETP and non-Tg) mice. Thus, this effect is independent of the lipemic phenotype and of the presence of the proteins apo CIII and CETP. However, cholesterol distribution in plasma lipoproteins was affected in a genotype dependent manner. Fibrates induced VLDL-chol increases and HDL-chol reductions only in apo CIII hypertriglyceridemic mice (CIII and CIII/CETP). Although ciprofibrate increased plasma CETP activity, the reduction in HDL-chol was independent of CETP, since it occurred also in the absence of this protein (CIII only mice). HDL is probably reduced because in rodents fibrates decrease apo AI synthesis [21]. The preservation of HDL-cholesterol levels in treated normotriglyceridemic CETP and non-Tg mice is probably associated to the decreased activity of HL observed in these treated mice. Staels et al. [47] also reported that fenofibrate induced reduction in HL. On the other hand, ciprofibrate induced reductions in LDL-chol only in the presence of CETP, either in normo- and in hypertriglyceridemic mice (CIII/CETP and CETP), whereas in mice that did not express CETP, the fibrate treatment increased LDL cholesterol levels (CIII and non-Tg).

Conflicting results have been reported regarding the effect of fibrate treatments on the plasma CETP activity. In the present study, we demonstrate that ciprofibrate induced elevation of CETP plasma protein and liver mRNA levels. The mechanism underlying the CETP gene activation is likely transcriptional, either directly through a putative PPAR response element in the CETP promoter [48], or through PPARα induction of LXR which, in its turn, enhances CETP gene transcription [49].

The opposite effect of fibrate in increasing CETP and reducing HL levels in CETP transgenic mice led us to suppose that the indirect reverse cholesterol transport would be facilitated in these treated mice. Indeed, we observed that higher amounts of ^3^H-HDL and lower ^3^H-LDL remained in the plasma of ciprofibrate treated CETP mice 6 hours after the ^3^H-HDL injection. Thus, ciprofibrate increased CETP mediated transfer of ^3^H-CEt from HDL to LDL and accelerated plasma removal of ^3^H-CEt-LDL. Since LDL receptor expression seems not to be affected by fibrates, it is conceivable to think that ciprofibrate, or the consequent elevated CETP activity, could improve LDL particle affinity for its receptor, and hence speed up LDL plasma removal process. Accordingly, Sakai et al [50] reported that CETP deficient patients presented a retarded plasma clearance of LDL particles because of a lower affinity for their receptor. Alternatively, fibrate stimulated LPL and repressed HL activities could have generated more IDL. Since IDL is removed from plasma much faster than LDL this would explain the lower levels of LDL. In addition, CETP, alone or in conjunction with HL, must play a critical role in either IDL or LDL removal process, since ciprofibrate did not lower the plasma LDL levels in non-transgenic mice, which also presented higher LPL and lower HL activities.

**Figure 3:**
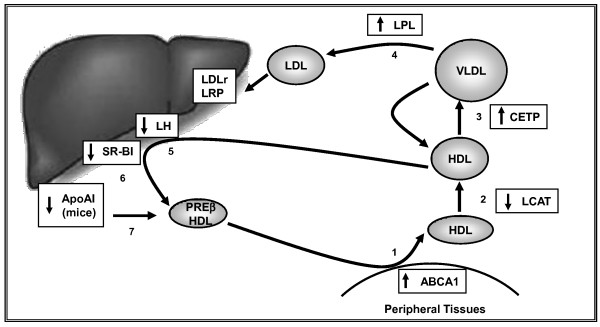
**Diagram showing increased indirect reverse cholesterol transport steps as a response to ciprofibrate treatment**. Changes in the steps 3, 4 and 5 were verified in the present work, and other steps were previously described for different fibrates and animal species, as follow: Step 1: Chinetti G, et al. Nat Med. 2001;7:53-58; Step 2: Staels et al., 1992 and Bouly et al [ref [23,47]]; Step 4: Berger & Moller, Annu Rev Med 2002;53:409-35; Step 5: Staels et al., 1992 [ref [47]]; Step 6: Mardones et al., 2003 [ref [51]]; Step 7: Staels et al., 1998 [ref [19]]. ABCA1: ATP-binding cassette transporter-A1; LCAT: lecithin cholesterol acyl transferase; LDLr: LDL receptor; LRP: LDL receptor related protein; SRB1:Scavenger receptor class B type I.

Higher CETP activity induced by ciprofibrate did not result in reduction of HDL-cholesterol because plasma removal of HDL was also impaired in fibrate treated mice. Besides reducing HL activity, fibrate treatment may have also diminished SRBI expression, as described by Mardones et al. [51]. Nonetheless, whole liver cholesterol uptake was increased in ciprofibrate treated CETP transgenic mice, suggesting that the indirect (through LDL) reverse cholesterol transport was more effective in CETP treated mice, as depicted in the diagram in figure 3.

## Conclusion

Together these data suggest that ciprofibrate treatment of human CETP expressing model is highly beneficial by improving the flow of cholesterol through the indirect reverse cholesterol transport system and preserving HDL particles in the plasma compartment to play their pleiotropic antiatherogenic actions. In humans, these effects could be even more positive considering that fibrates also increase apo AI synthesis.

## List of abbreviations

**Apo**: apolipoprotein, **CE**: cholesteryl ester, **CEt**: cholesteryl ether, **CHD**: coronary heart disease, **chol**: cholesterol, **FFA**: free fatty acids, **FPLC**: fast protein liquid chromatography, **HL**: hepatic lipase, **LPL**: lipoprotein lipase, **PPAR**: peroxissome proliferator activated receptors, **TG**: triglycerides.

## Competing interests

The authors declare that they have no competing interests.

## Authors' contributions

EJBB and PRP equally participated in all experiments, analysis and data interpretation and helped to draft the manuscript. ACC and JAB carried out mice breeding and screening and helped with plasma biochemical analysis and CETP activity assay. HCFO conceived the study and its design, obtained research grants for its development, supervised all technical activities, coordinated data interpretation and wrote the final version of the manuscript. All authors read and approved the final manuscript.
